# Lattice-shaped catheter ablation for incessant inducible AVNRT in patient undergoing atrial fibrillation ablation: a case report

**DOI:** 10.1093/ehjcr/ytag221

**Published:** 2026-03-18

**Authors:** Alhasan Alzubi, Mohamed Shelig, Abdulmueti Alhadi, Ashraf M Elghul, Khalid Abozguia

**Affiliations:** Department of Medicine, Marshall University Joan C. Edwards School of Medicine, 1600 Medical Center Drive, Huntington, WV 25701, USA; Department of Medicine, Marshall University Joan C. Edwards School of Medicine, 1600 Medical Center Drive, Huntington, WV 25701, USA; Mayo Clinic, Division of Nephrology & Hypertension, 200 First St. SW, Rochester, MN 55905, USA; Department of Medicine, Marshall University Joan C. Edwards School of Medicine, 1600 Medical Center Drive, Huntington, WV 25701, USA; Division of Cardiology, Marshall University Joan C. Edwards School of Medicine, 1600 Medical Center Drive, Huntington, WV 25701, USA

**Keywords:** Pulsed Field Ablation, Sphere-9 Catheter, Atrial Fibrillation Ablation, Lattice-Tip Catheter, Case Report

## Abstract

**Objective:**

This report describes the first use of the Affera Sphere-9 dual-energy catheter for slow pathway modification in atrioventricular nodal re-entrant tachycardia (AVNRT) during atrial fibrillation (AF) ablation in a patient with a dual-chamber pacemaker, illustrating a streamlined single-catheter approach.

**Case presentation:**

A 74-year-old man with prior cryoablation for AF, mitral valve repair, and a recently implanted dual-chamber pacemaker was referred for repeat AF ablation due to symptomatic, recurrent AF (30% burden). Left atrial mapping with the Sphere-9 catheter revealed reconnection at the right superior pulmonary vein, right carina, and across prior roof and floor lines. Using pulsed field (4-s lesions, 8 mm spacing) and supplemental radiofrequency (RF) energy, the left atrial posterior wall was re-isolated. Cavo-tricuspid isthmus ablation was performed with RF and pulsed field applications, achieving bidirectional block. During subsequent testing, typical AVNRT was induced. To maximize efficiency, slow pathway modification was performed with the same Sphere-9 catheter, using shortened RF applications (3 s), a lower target temperature (60°C), and an 80% current limit, at an inferior-posterior site 1.3–2.5 cm from the His signal. Junctional acceleration was observed without atrioventricular block, indicating successful slow pathway modification. The procedure was completed without fluoroscopy or complications, and follow-up device interrogation showed no recurrence of AF or AVNRT.

**Conclusion:**

This case demonstrates the feasibility and acute safety of using a single dual-energy lattice-tip catheter for combined AF and AVNRT ablation, potentially reducing procedure time, catheter exchanges, and cost; larger studies are needed to assess long-term outcomes.

Learning points

- A single lattice-tip mapping/ablation catheter capable of RF and pulsed-field energy may streamline complex ablation workflows by reducing catheter exchanges.
- Slow-pathway modification for typical AVNRT can be performed safely with conservative dosing and careful anatomic targeting to minimize AV node injury risk.
- Junctional beats during slow-pathway ablation provide real-time feedback of effective lesion delivery and help guide procedural endpoints.

## Introduction

Atrial fibrillation (AF) is the most common sustained arrhythmia and a major driver of stroke, heart failure, and health-care utilization. Thermal ablation (point-by-point radiofrequency or cryo) is effective but can be limited by prolonged procedure times and risks of collateral thermal injury (oesophagus, phrenic nerve). Pulsed field ablation (PFA) delivers microsecond, high-voltage electric pulses that produce myocardial-selective lesions via irreversible electroporation, enabling efficient pulmonary vein isolation (PVI) with a distinct safety profile compared with heat.^1^ Randomized data in paroxysmal AF demonstrate non-inferiority of PFA to conventional thermal ablation, and large registries describe a low overall complication rate with a different spectrum of events than thermal energy.^2,3^

First-generation multielectrode pentaspline PFA balloons are optimized for ‘single-shot’ PVI, yet their large footprint and fixed spacing are less suited to dense mapping or focal extra-PVI work.^2^ A newer dual-energy lattice-tip platform, the Sphere-9 catheter used with the Affera mapping/ablation system—integrates high-density mapping, pacing, and both PFA and RF delivery on a single catheter. In persistent AF, this approach was non-inferior to conventional RF for safety and effectiveness while streamlining ‘map-and-ablate’ workflow.^4^ The focal lattice-tip design also supports on-the-fly switching between PFA and RF to tailor lesion sets to anatomic context^5^ . Importantly, early data suggest PFA can be performed safely in patients with cardiac implantable electronic devices (CIEDs) when standard programming/surveillance is used, which is relevant when working near conduction tissue in device carriers.^6,7^

## Summary figure

**Figure:**
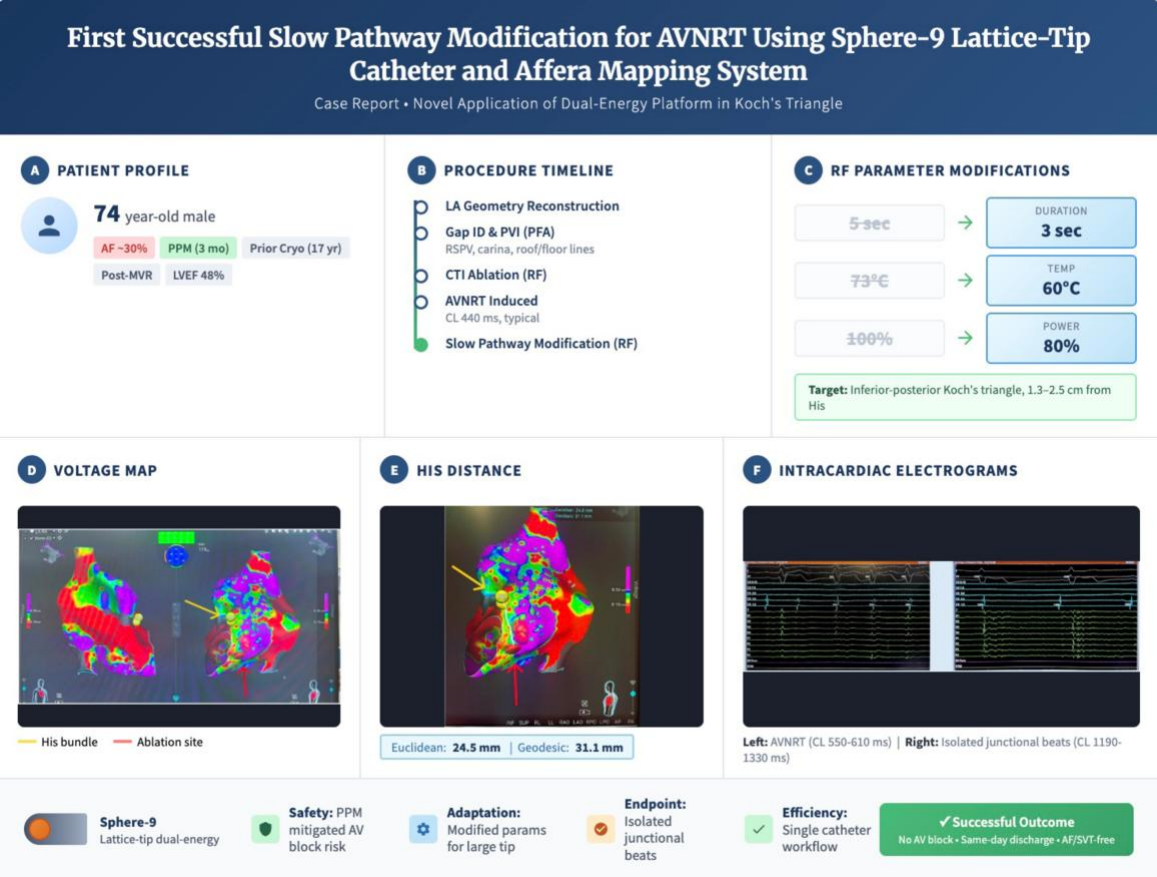


## Case presentation

A 74-year-old man with AF, status post cryoballoon ablation 17 years earlier; mitral valve prolapse, status post-surgical repair 18 years earlier; tachy-brady syndrome with a dual-chamber permanent pacemaker implanted 3 months earlier; hypertension; hyperlipidemia; and chronic kidney disease, was referred for repeat AF ablation. He was taking oral anticoagulation and metoprolol 50 mg twice daily. Pacemaker interrogation showed an AF burden of ∼30% with persistent fatigue and presyncope with an otherwise unremarkable physical examination. Transthoracic echocardiography demonstrated a left ventricular ejection fraction of 48% with elevated filling pressures and a TAPSE of 1.63 cm.

In the electrophysiology laboratory, left atrial geometry was reconstructed using the Sphere-9 catheter and Affera mapping system.^4,5^ Conduction gaps were identified at the right superior pulmonary vein (RSPV) and the right pulmonary vein carina, with additional gaps across prior linear lesions at the mid roof line and mid floor line.

Pulmonary vein isolation (PVI) was performed using pulsed field ablation (PFA) with the Sphere-9 catheter to perform segmental isolation of the RSPV and right carinaas, as well as complete roof and floor line of posterior wall of LA. After ablation and a waiting period, entrance and exit block were confirmed in all PVs. Posterior wall of LA and all PVs remained electrically isolated from the left atrium. Cavotricuspid isthmus (CTI) ablation was then performed using the same Sphere-9 platform (radiofrequency energy), and bidirectional CTI block was confirmed by differential pacing.

Subsequent comprehensive electrophysiology testing revealed easily inducible typical atrioventricular nodal re-entrant tachycardia (AVNRT) (cycle length 440 ms),^8^ reproducibly initiated by catheter manipulation or atrial pacing and terminated by overdrive atrial pacing—findings consistent with typical AVNRT. The patient had no prior clinical documentation of AVNRT before the procedure. Intracardiac electrograms recorded during induction and ablation are shown in *Figure 2*.

**Figure 1:**
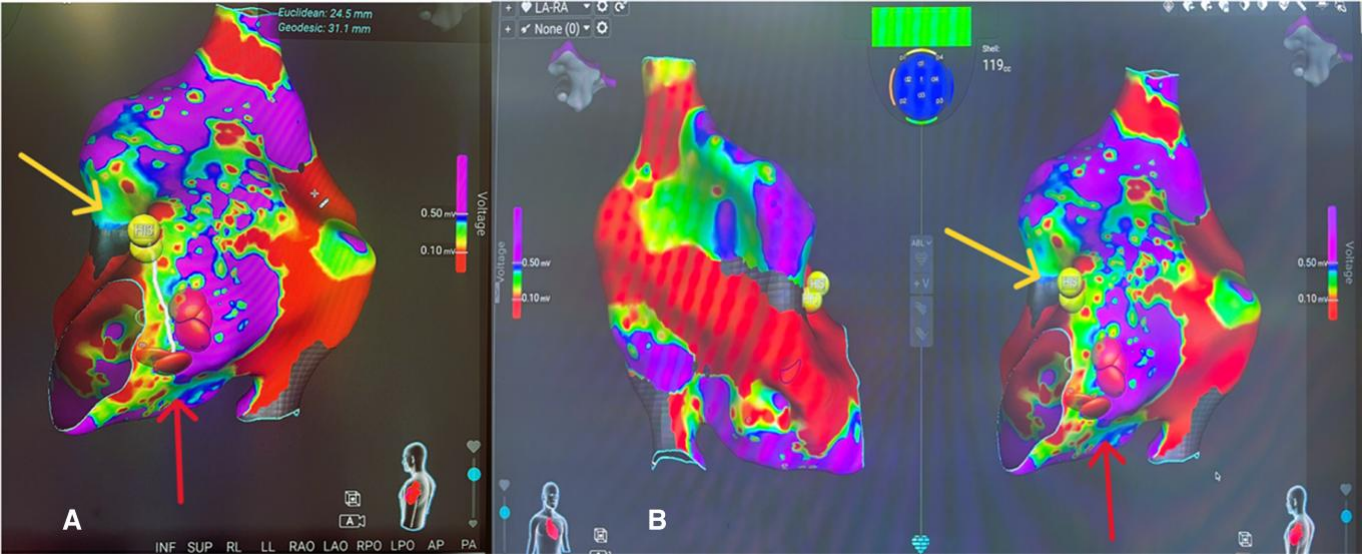
Right atrial voltage maps acquired with the Sphere-9/Affera system. (*A*) Voltage map demonstrating the His region (yellow arrow) at the superior border of Koch’s triangle and the slow-pathway ablation site (red arrow) placed inferior–posterior to the His (≈1.3–2.5 cm offset) to maximize AV nodal safety. (*B*) Additional right atrial projections confirming the same landmarks, with voltage scale set between 0.10 and 0.50 mV. Ablation at the marked site using short RF applications (3 s, 60°C, reduced power) produced isolated junctional beats and successful slow-pathway modification without AV block.

**Figure 2:**
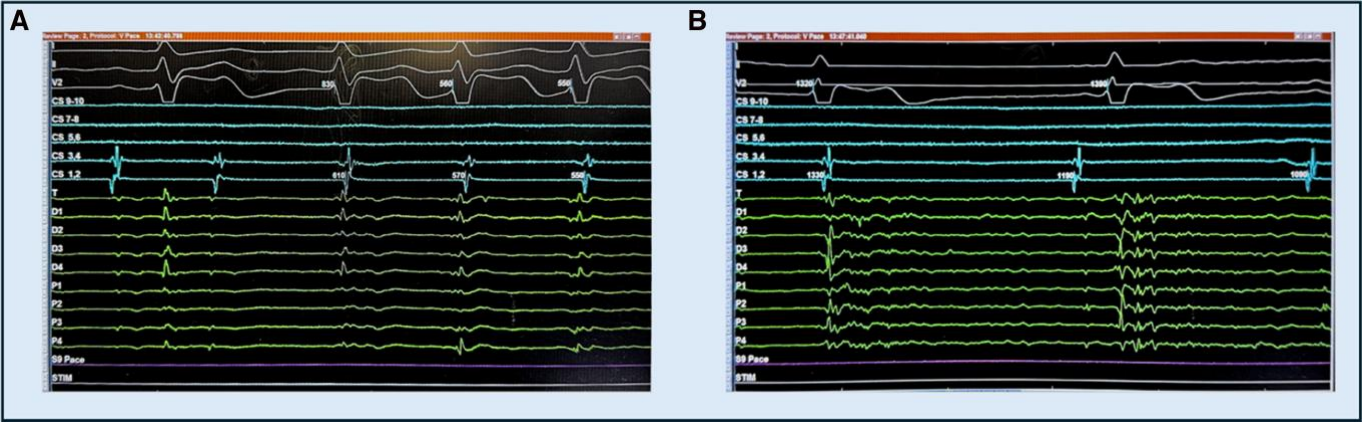
EGM tracing demonstrating induction of atrioventricular nodal re-entrant tachycardia (AVNRT) and successful slow pathway ablation. Leads I, II, V2 represent the surface EKG. Leads CS 9–10 through CS 1,2 represent our decapolar catheter positioned in the right atrium, and the Sphere-9 lattice-tip catheter is positioned at the tricuspid annulus (Leads T, D1–D4, P1–P4). (*A*) The first beat is sinus rhythm, followed by a premature atrial contraction with an AH jump and initiation of typical AVNRT. During this tachycardia, the VA interval is zero with the atrial signal coinciding with the surface QRS, characteristic of slow-fast AVNRT. (*B*) A junctional beat observed during radiofrequency ablation at the successful slow pathway site, followed by return of sinus rhythm. Junctional beats were not observed during prior unsuccessful ablation lesions. Following this brief application, AVNRT was no longer inducible.

A slow pathway modification was performed for AVNRT using a large Sphere-9 catheter to improve efficiency and cost-effectiveness. The patient’s pre-existing pacemaker mitigated the heightened risk of AV heart block associated with the larger catheter. Radiofrequency (RF) application parameters were adjusted to compensate for the larger electrode size, with the duration shortened to 3 s (from 5), the target temperature lowered to 60°C (from 73°C), and the power limited to 80%. Ablation was successfully performed in Koch’s triangle, targeting a slightly inferior-posterior site (1.3–2.5 cm from the His signal) to account for the larger tip.^9^ This resulted in isolated junctional beats and successful slow pathway modification^10^ (*Figure 1*). These conservative RF adjustments were made at operator discretion with safety as the priority, maintaining ≥20 mm distance from the His region and with the option to titrate settings upward if needed. An isolated junctional beat was observed during RF delivery at the successful site (*Figure 2*). After ablation, AVNRT was no longer inducible.

Post-procedure pacemaker interrogation was satisfactory with unchanged sensing and capture thresholds. The patient was discharged the same day on home medications and, on follow-up, has remained free of AF and SVT recurrence.

## Discussion

To our knowledge, this is the first published case of successful slow pathway modification ablation for typical AVNRT in patient undergoing AF ablation using the Affera mapping system and Sphere-9 lattice-tip dual-energy catheter.

In this patient, we elected to perform slow-pathway modification for AVNRT using a large Sphere-9 catheter, an approach not typically applied in Koch’s triangle setting (*Figure 1*). The presence of a pre-existing pacemaker in this patient offered a degree of safety, reducing the clinical consequences of inadvertent AV nodal injury and allowing us some flexibility in technique.^8^ Importantly, AVNRT had not been clinically documented prior to the procedure and was identified intraprocedurally during electrophysiology testing (*Figure 2A*). A key limitation in this case was the unexpectedly easy inducibility of typical AVNRT, which was not anticipated pre-procedure and introduced additional procedural complexity. At the time of the procedure, published experience describing slow pathway modification using a large-footprint focal lattice-tip catheter was limited, largely due to concern for inadvertent complete heart block. Because the patient already had a pacemaker, we proceeded with a deliberately conservative strategy.

Because a larger catheter tip carries inherent risks (especially of excessive heating, broader lesion spread, or unintended conduction system injury), we adjusted the standard RF settings. We shortened the ablation duration from 5 to 3 s, lowered the target temperature from 73°C to 60°C, and limited the power to 80% of the usual setting. These parameter changes were intended to reduce thermal overload and lateral spread of injury, while still delivering sufficient energy to ablate the slow-pathway substrate. Moreover, recognizing the increased tip radius, we deliberately targeted a slightly inferior-posterior region (1.3–2.5 cm from the His signal) rather than the classic locus—effectively increasing the separation from the compact AV node. These conservative adjustments were made at operator discretion with safety as the priority, with the understanding that settings could be titrated upward if needed. We also intentionally maintained ≥20 mm separation from the His region to increase safety given the catheter footprint.

During ablation, isolated junctional beats emerged (*Figure 2B*) rather than slow junctional beat observed routinely during conventional ablation with conventional small size tip ablation catheter. This is explained by the fact that our RF application is short in time (3 s) compared to 30–60 s with convention aRF catheter. A practical constraint is the system-imposed maximum RF lesion duration (7 s), which limits the ability to deliver conventional longer RF applications in this anatomic region.

Isolated junctional beats were observed at successful site which is a favourable sign that the slow pathway was engaged without causing direct AV nodal injury. The end-point of slow pathway modification was achieved without occurrence of AV block, underscoring that the tailored approach was effective acutely in this particular patient. The successful outcome suggests that, under appropriate conditions, a large-tip catheter may be feasible in carefully selected cases when energy delivery is deliberately conservative. Because the Sphere-9 platform can deliver both RF and pulsed field ablation (PFA), energy selection is relevant; we chose RF for slow pathway modification given its established clinical experience and familiar intraprocedural end-points, whereas experience with PFA for AVNRT remains limited.^11,12^ Transient atrioventricular block and slow junctional rhythm have been reported during pulsed field ablation for slow pathway modification, warranting caution near the His/AV nodal region.^13^ PFA can also introduce electrical noise on intracardiac electrograms, which may complicate real-time interpretation of junctional activity during ablation.

Nevertheless, several caveats warrant emphasis. This is a single case, and we cannot infer general safety or effectiveness without broader experience. Also, overconservative parameter reduction might risk suboptimal lesion formation and higher recurrence rates.^3,7^ Long-term follow-up is essential to rule out late conduction injury or arrhythmia recurrence.^8^ Finally, operator experience is critical: Precise catheter manipulation, real-time feedback on contact, temperature control, and anatomical awareness are all prerequisites to success in such a modified technique.

## Conclusion

In conclusion, this case demonstrates procedural feasibility of performing slow pathway modification for typical AVNRT using the Sphere-9 lattice-tip dual-energy catheter during AF ablation when AVNRT is encountered intraprocedurally. Given the single-case nature of this report, broader studies are required to define safety, optimal RF/PFA strategies, and longer-term outcomes for slow pathway modification with this catheter platform.

## Lead author biography


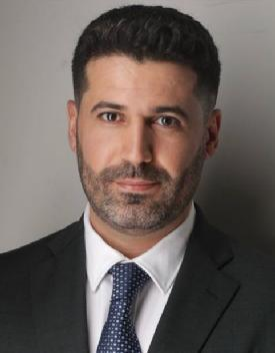


Dr Alhasan Alzubi is an Internal Medicine resident at Marshall University Joan C. Edwards School of Medicine in Huntington, West Virginia, USA. He serves as research chief for the Internal Medicine department and is actively involved in clinical and translational cardiovascular research. His main interests include cardiac electrophysiology, atrial fibrillation ablation, structural heart disease, and outcomes research using large databases and meta-analyses. He has presented multiple abstracts at regional and national cardiology meetings and aims to pursue a career in academic cardiology. Outside of medicine, he enjoys teaching trainees and collaborating on multidisciplinary research projects.

**Consent:** The authors confirm that witnessed verbal consent for submission and publication of this case report including images and associated text has been obtained from the patient. This has been discussed with the editors.

## Data Availability

All relevant data supporting the findings of this case report are included within the article. No additional data are available due to patient privacy and confidentiality. This material has not been published in any meeting or journal. Our institution does not require ethical approval for reporting individual cases or case series.
